# Changes in community mental health services availability and suicide mortality in the US: a retrospective study

**DOI:** 10.1186/s12888-020-02607-y

**Published:** 2020-04-25

**Authors:** Peiyin Hung, Susan H Busch, Yi-Wen Shih, Alecia J McGregor, Shiyi Wang

**Affiliations:** 1 Department of Health Services Policy and Management, Arnold School of Public Health, University of South Carolina, 915 Greene Street, Suite 348, Columbia, SC 29201 USA; 2 Department of Health Policy and Management, Yale University, School of Public Health, 60 College Street, Suite 300B, New Haven, CT 06510 USA; 3 Department of Community Health, Medford, Tufts University, School of Arts and Sciences, 574 Boston Avenue, Suite 208, Medford, MA 02155 USA; 4 Department of Chronic Diseases Epidemiology, Yale University, School of Public Health, 60 College Street, Suite 432, New Haven, CT 06510 USA

**Keywords:** Suicide, Deinstitutionalization, Access to mental health care, Community mental health

## Abstract

**Background:**

Despite the fact that the overwhelming majority of mental health services are delivered in outpatient settings, the effect of changes in non-hospital-based mental health care on increased suicide rates is largely unknown. This study examines the association between changes in community mental health center (CMHC) supply and suicide mortality in the United States.

**Methods:**

Retrospective analysis was performed using data from National Mental Health Services Survey (N-MHSS) and the Centers for Disease Control and Prevention (CDC) Wide-Ranging Online Data for Epidemiologic Research (WONDER) (2014–2017). Population-weighted multiple linear regressions were used to examine within-state associations between CMHCs per capita and suicide mortality. Models controlled for state-level characteristics (i.e., number of hospital psychiatric units per capita, number of mental health professionals per capita, age, race, and percent low-income), year and state.

**Results:**

From 2014 to 2017, the number of CMHCs decreased by 14% nationally (from 3406 to 2920). Suicide increased by 9.7% (from 15.4 to 16.9 per 100,000) in the same time period. We find a small but negative association between the number of CMHCs and suicide deaths (− 0.52, 95% CI − 1.08 to 0.03; *p* = 0.066). Declines in the number of CMHCs from 2014 to 2017 may be associated with approximately 6% of the national increase in suicide, representing 263 additional suicide deaths.

**Conclusions:**

State governments should avoid the declining number of CMHCs and the services these facilities provide, which may be an important component of suicide prevention efforts.

## Background

Suicide is the 10th leading cause of death in the U.S., claiming over 40,000 lives annually [1]. From 1999 to 2017, the average age-adjusted mortality rate attributable to suicide in the U.S. increased from 10.5 to 14.0 deaths per 100,000 [1]. This is in contrast to suicide rates in other Organization for Economic Co-operation and Development (OECD) countries, which fell by more than 10% during 1999–2015 [2]. This dramatic increase in suicide has led some to speculate that reductions in hospital-based psychiatric beds may have contributed to the rise in suicides [3–6]. Yet, the most recent evidence indicates that, within states, changes in the number of psychiatric beds was not associated with changes in suicide rates [3].

Somewhat surprisingly, few studies have considered whether changes in access to non-hospital-based mental health care is associated with increased suicide, even though the overwhelming majority of mental health services are delivered in an outpatient setting [7]. In the U.S. under the 1963 Community Mental Health Act (CMHA) [8], care for individuals with mental health disorders dramatically moved away from inpatient care with the goal of providing treatment in less restrictive settings. Yet, due to a lack of funding, psychiatric hospital closures were not accompanied by adequate increases in community-based treatment [9–11]. Today, although evidence-based practices that have been shown to improve symptoms of those with serious mental illness exist, few individuals receive these services [12, 13]. In addition, many individuals in need do not receive any treatment [14]. Among individuals with serious thoughts of suicide, only about half received mental health treatment in the past year [15].

Recent anecdotal reports have brought attention to the effects of CMHC closures on access to care for individuals with serious mental illness [16–18]. For low-income individuals, outpatient mental health facilities are often the only available specialty treatment in their community [9]. CMHCs provide a range of specialized services for individuals with serious mental illness, as well as routine care for patients who have been discharged from an inpatient mental health facility [7]. In particular, among different types of outpatient mental health settings, CMHCs were the most likely to provide suicide prevention services, psychiatric emergency walk-in services, case management, crisis intervention treatments, and to accept patients across all ages [7]. The combination of these services provided at CMHCs may be an integral part of mental health care to individuals considering suicide [7].

Given the disturbing trend of suicide deaths in the U.S., understanding whether the availability of CMHCs is associated with suicides can inform the current dialogue on how best to allocate limited public dollars to facilitate suicide prevention. This study fills the evidence gap by examining changes in the number of CMHCs in the U.S. per capita, and whether and how changes in number of CMHCs may have played a role in changes in suicide mortality.

## Methods

### Data and study population

We used state-level data on the number of hospital-based psychiatric facilities, residential care settings, CMHCs, partial hospitalization/day treatment settings for the years 2014–2017 for 50 U.S. states and the District of Columbia (DC) from the National Mental Health Services Survey (N-MHSS) [7]. The N-MHSS is an annual survey that collects information from all known facilities providing mental health services in the U.S., including psychiatric hospitals, nonfederal general hospitals with separate psychiatric inpatient units, CMHCs, and partial hospitalization/day treatment facilities. All facilities reported their treatment characteristics during the survey, including settings of care (inpatient, residential, partial hospitalization/day treatment, or outpatient) and the provision of suicide prevention services. One objective of the N-MHSS is to update SAMHSA’s inventory of all known mental health and substance abuse treatment facilities. To our knowledge, this is the only comprehensive source of national data on specialty mental health facilities and their scope of clinical services. The N-MHSS began distinctly identifying federal- and state-licensed CMHCs in 2014. In all four study years, N-MHSS excluded mental health facilities that were 1) Department of Defense military treatment facilities, 2) individual private practitioners or small group practices not licensed as a mental health clinic or center; and 3) facilities in jails or prisons [7]. All licensed psychiatric hospitals, hospitals with inpatient psychiatric units, residential care settings, and CMHCs (including partial hospitalization/day treatment settings) that meet state licensing or certification requirements are eligible for inclusion in the survey. Mental health facilities that have closed since the previous-year survey are excluded. During the study period, response rates were 88.1% of 16,687 eligible facilities in 2014; 91.9% of 14,573 eligible facilities in 2015; 91.1% of 13,983 eligible facilities in 2016; and 93.0% of 13.618 eligible facilities in 2017, with item response rates averages of 96.9, 97.9, 97.6, and 98%, respectively [7].

Data on state-level mental health professionals came from the Occupational Employment Statistics 2014–2017, which produced employment estimates for 415 industry classifications by state. Based on the North American Industry Classification System, occupations related to mental health care include a) psychiatrists, b) psychiatric technicians, c) psychiatric aides, d) clinical, counseling, and school psychologists, e) all other psychologists, f) mental health counselors, h) mental health and substance abuse social workers [19]. Detailed definitions for each professional can be found in the US Bureau of Labor Statistics [19]. In particular, psychiatric technicians and aides are certified to have the privilege caring for people who have mental illness.

State-level annual suicide mortality was derived from the Centers for Disease Control and Prevention’s (CDC) Wide-Ranging Online Data for Epidemiologic Research (WONDER). For each state in each year (2014–2017), we considered population size and number of deaths from suicides (intentional self-harm; ICD-10-CM diagnosis codes U03, X60-X71, X72-X74, X75-X84, and Y87.0). All 50 states and the District of Columbia had at least 35 suicide deaths in each year of our study.

### Measures

#### Community mental health centers and hospital-based psychiatric care settings

N-MHSS respondents were asked which of the following categories best describe their facility^7^: 1) psychiatric hospital, 2) separate inpatient psychiatric unit of a general hospital, 3) residential treatment center for children, 4) residential treatment center for adults, 5) other type of residential treatment facility, 6) Veterans Administration Medical Center or other VA health care facility, 7) Community mental health center (CMHC), 8) Outpatient mental health facility, 9) Multi-setting mental health facility (nonhospital residential plus either outpatient and/or partial hospitalization/day treatment). This study focused on CMHCs because these facilities were more likely to accept uninsured or Medicaid-insured patients, to offer suicide prevention services, psychiatric emergency walk-in services, case management, and other specialty practices, compared to other outpatient or multi-setting facilities (Appendix Table 1). N-MHSS respondents received a link of descriptions of each facility type [7]. A CMHC was defined as a facility that provided any of the following services: 1) outpatient services, 2) 24-h emergency care services, 3) day treatment or other partial hospitalization services, or psychosocial rehabilitation services, and 4) screening for inpatient services to state mental health facilities, and that met applicable licensing or certification requirements for community mental health centers in a state where it is located. Beginning in 2015, a new category, “partial hospitalization/day treatment facility” was added, leading to a separate category for CMHCs to choose, should a CMHC primarily focuses on partial hospitalization/day treatment services [7]. In addition to the self-reported CMHC status, non-hospital mental health facilities that reported providing both outpatient services and day treatment or other partial hospitalization services were also categorized as a CMHC in this study. Using the total number of CMHC/partial hospitalization/day treatment facilities (hereafter called CMHCs) in conjunction with U.S. Census state population estimates, we calculated the number of CMHCs per 100,000 persons in each year-state.

To address changes in the hospital-based inpatient psychiatric supply by state, we considered hospital-based psychiatric services in all regressions. When determining the availability of hospital-based psychiatric settings, we calculated the number of psychiatric hospitals or separate inpatient psychiatric units of a general hospital per 100,000 persons each year.

#### Mental health professional supply

Overall changes in the supply of individual mental health professionals in each state per year are essential for suicide prevention and for facility provision services, as difficulty in staffing may result in facility closures. Thus, in all models, we also included state-level number of psychiatrists, psychiatric technicians, psychiatric aides, clinical, counseling, and school psychologists, all other psychologists, mental health counselors, or mental health and substance abuse social workers, per 100,000 persons.

#### Covariates

In accordance with variables described in previous literature [4, 6, 20], we calculated the following covariates using U.S. Census Bureau data to control for relevant population-level characteristics: *percent population by age group* (less than 15 years old, 15–24 years old, 25–44 years old, 45–64 years old, 65–74 years old, 75 years old or more); *percent race/ethnicity* (White Non-Hispanic, Black/African American Non-Hispanic, Hispanic, Asian, American Indian and Alaska Native, Native Hawaiian or other Pacific Islander, and multiple race individuals); *percent of state residents below 200% Federal Poverty Levels (FPL).*

### Statistical analysis

We first visually plotted mean state CMHCs rates and mean state suicide rates pooled over 4 years (2014–2017) to better understand the cross-sectional associations between these variables.

Because state availability of CMHCs and hospital-based psychiatric units varied substantially across states and from 2014 to 2017, we used state-level variations in the timing and size of changes in CMHCs and hospital-based psychiatric units per 100,000 persons to identify the independent associations of changes in the supply of CMHCs and hospital-based psychiatric units with suicide rates, controlling for state-level mental health professionals per 100,000 persons in each year.

We used multivariate generalized linear time series models to analyze changes in state suicide mortality and sequentially included state and year fixed effects, as well as sociodemographic, and socioeconomic characteristics in a series of models. To identify possible multicollinearity between covariates, we used variance inflation factors (VIFs) [21]. The state-level proportion of population below 200% FPL was highly collinear (VIF > 10) with other socio-economic factors (proportion of population that was unemployed and proportion of individuals younger than 65 without health insurance), as were unemployment rates and uninsured rates among population ages < 65. Therefore, we controlled for these factors in separate multivariate models, in addition to unobservable characteristics unique to each year and each state by having year and state fixed effects.

In all models, we weighted observations by state-year population. This also accounts for state heteroscedasticity, as variability in suicide rates may be inversely correlated with state population. Given the time-sequenced nature of the suicide data, all models generated robust standard errors, accounting for intra-state correlated variances across years, to adjust for possible residual autocorrelations.

The final model included four observations for each state, and included the state-year number of CMHCs per 100,000 persons, number of hospitals with psychiatric services per 100,000 persons, number of mental health professionals per 100,000 persons, percent population below 200% FPL, percent population by age group, percent population by race/ethnicity, state and year fixed effects. Finally, to better illustrate within-state association between the availability of CMHCs and suicide rates, we graphed the adjusted suicide mortality rates for each state-year against availability of CMHCs in the analogous state-year, linking the four data points from each state.

All analyses were conducted using SAS (version 9.4), and Stata (version 15); *p*-values < .05 were considered as statistically significant. The Institutional Review Board at the authors’ University designated this study exempt from review.

## Results

### National Changes in number of community mental health centers and suicide rates

**Fig. 1:**
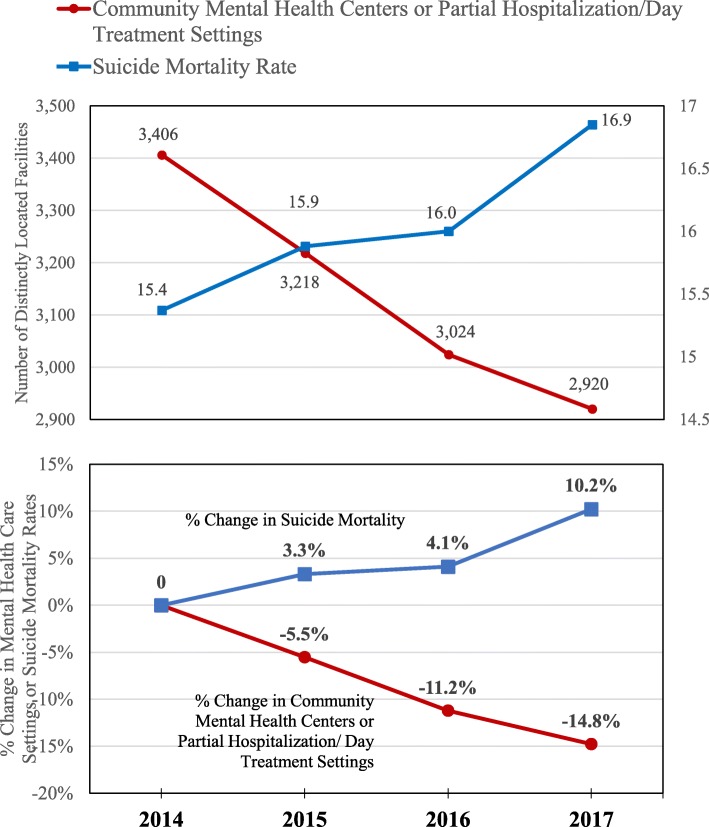
Trend of Mental Health Facilities Supply and Suicide Mortality in the United States 2014–2017. NOTE Data were from 2014 to 2017 National Mental Health Services Survey (N-MHSS) to identify state-level community mental health center status during 2014–2017 and from Centers for Disease Control and Prevention (CDC) Wide-Ranging Online Data for Epidemiologic Research (WONDER) data for suicide rates, identified as intentional self-harm by ICD-10-CM diagnosis codes U03, X60-X71, X72-X74, X75-X84, Y87.0. A Community Mental Health Center was defined as a facility that provided any of the following services: 1) outpatient services, 2) 24-h emergency care services, 3) day treatment or other partial hospitalization services, or psychosocial rehabilitation services, and 4) screening for inpatient services to state mental health facilities, and that met applicable licensing or certification requirements for community mental health centers in a state where it is located [7]. Mental health care facilities self-identified as a partial hospitalization or day treatment facility were also categorized as a community mental health center

Figure 1 shows national changes in the number of CMHCs and suicide mortality rates during the period from 2014 to 2017. When only looking at the annual trend across all states, the number of CMHCs decreased from 3406 to 2920 while suicide mortality rates increased from 15.37 to 16.85 per 100,000 persons. For the 4 years from 2014 to 2017, these changes amounted to a 14.27% decrease in the number of CMHCs and a 9.63% increase in suicide mortality rates.

### State-level changes in community mental health centers and suicide

National data obscures state variation in both numbers of CMHCs and changes in suicide rates. Between 2014 and 2017, over two-thirds of states experienced decreased numbers of CMHCs (Table 1). Percentage changes in the number of CMHCs ranged from − 58.3% in Alaska to + 92.86% in Wyoming. Despite the increasing trend in suicide rates nationally, 6 states have improved suicide mortality rates between 2014 and 2017; still, changes in suicide mortality rates varied substantially by state with percentage changes ranging from − 18.9% in DC to + 32.0% in South Dakota. Of all 50 states and DC, 33 (64.7%) simultaneously experienced a decrease in the number of CMHCs and an increase in suicide mortality rates between 2014 and 2017. In contrast, several states – CA, DC, DE, NJ, NY – had significant decreases in the number of CMHCs without a concurrent increase in suicide mortality rates. Similarly, some states –SC, SD, TN, TX, UT, VA, WA, WI, WV, WY – had an increased number of CMHCs but still experienced higher suicide rates. Per capita, there was a 16-fold variation in the number of CMHCs across states in 2014 (Appendix Table 2), ranging from 0.37 per 100,000 in North Carolina to 5.86 per 100,000 in Montana in 2014. Between 2014 and 2017, this variation dramatically increased to 25-fold, when the number of CMHCs per 100,000 decreased in most states (*n* = 40; 78.4%).

**Table 1:** Change in the Number of Community Mental Health Centers and Suicide Mortality Rates by State, 2014–2017

	Number of Community Mental Health Centers			Suicide Mortality Rates		
	2014	2017	% Change 2014–2017	2014	2017	% Change 2014–2017
Nationwide	3406	2920	−14.27%	15.37	16.85	9.63%
AK	36	29	−19.44%	22.67	27.03	19.25%
AL	78	70	−10.26%	14.74	17.15	16.35%
AR	91	80	−12.09%	17.5	21.00	20.02%
AZ	55	32	−41.82%	18.6	18.91	1.68%
CA	157	104	−33.76%	10.99	10.91	−0.76%
CO	82	89	8.54%	20.41	21.06	3.20%
CT	31	34	9.68%	10.57	11.29	6.78%
DC	7	7	0.00%	8.35	6.77	−18.89%
DE	8	7	−12.50%	13.47	11.64	− 13.56%
FL	156	101	−35.26%	15.35	15.38	0.18%
GA	65	41	−36.92%	12.91	13.91	7.77%
HI	15	8	− 46.67%	14.58	15.90	9.06%
IA	65	60	−7.69%	13.13	15.23	15.97%
ID	46	25	−45.65%	19.58	22.83	16.61%
IL	146	101	−30.82%	10.97	11.51	4.96%
IN	154	139	−9.74%	14.39	16.38	13.83%
KS	77	73	−5.19%	15.74	18.98	20.60%
KY	113	103	−8.85%	16.52	17.29	4.64%
LA	29	15	−48.28%	14.67	15.37	4.77%
MA	45	39	−13.33%	8.88	9.94	11.96%
MD	76	37	−51.32%	10.39	10.41	0.19%
ME	31	27	− 12.90%	16.54	20.51	24.00%
MI	105	100	−4.76%	13.72	14.63	6.60%
MN	69	60	−13.04%	12.64	14.04	11.08%
MO	58	65	12.07%	16.89	18.83	11.47%
MS	109	89	−18.35%	12.83	14.91	16.23%
MT	60	44	−26.67%	24.52	29.61	20.74%
NC	37	29	−21.62%	13.71	14.81	7.99%
ND	4	7	75.00%	18.53	20.39	10.02%
NE	14	27	92.86%	13.39	14.32	6.96%
NH	27	31	14.81%	18.62	19.73	5.99%
NJ	91	82	−9.89%	8.91	8.83	−0.92%
NM	29	22	−24.14%	21.63	23.51	8.71%
NV	12	5	−58.33%	20.22	20.91	3.43%
NY	150	110	−26.67%	8.72	8.54	−2.01%
OH	210	168	−20.00%	12.95	14.92	15.25%
OK	64	59	−7.81%	19.06	19.23	0.90%
OR	43	35	−18.60%	19.75	19.91	0.83%
PA	107	88	−17.76%	14.3	15.85	10.86%
RI	23	17	−26.09%	10.8	12.17	12.72%
SC	50	59	18.00%	15.67	16.68	6.44%
SD	26	25	−3.85%	16.64	21.96	31.99%
TN	104	86	−17.31%	15.25	17.36	13.85%
TX	109	124	13.76%	12.16	13.35	9.77%
UT	31	35	12.90%	19.03	21.37	12.32%
VA	87	87	0.00%	13.55	13.92	2.73%
VT	27	25	−7.41%	19.79	17.96	−9.25%
WA	89	113	26.97%	15.97	17.51	9.66%
WI	37	29	−21.62%	13.37	15.98	19.51%
WV	43	55	27.91%	19.73	21.64	9.69%
WY	28	23	−17.86%	20.54	27.10	31.94%

SOURCE Data were from 2014 to 2017 National Mental Health Services Survey (N-MHSS) to identify state-level mental health facility status during 2014–2017 and from Centers for Disease Control and Prevention (CDC) Wide-Ranging Online Data for Epidemiologic Research (WONDER) data for suicide rates, identified as intentional self-harm by ICD-10-CM diagnosis codes U03, X60-X71, X72-X74, X75-X84, Y87.0. NOTE A Community Mental Health Center was defined as a facility that provided any of the following services: 1) outpatient services, 2) 24-h emergency care services, 3) day treatment or other partial hospitalization services, or psychosocial rehabilitation services, and 4) screening for inpatient services to state mental health facilities, and that met applicable licensing or certification requirements for community mental health centers in a state where it is located [7]. Mental health care facilities self-identified as a partial hospitalization or day treatment facility were also categorized as a community mental health center

### Between-state association between community mental health centers and suicide

**Fig. 2:**
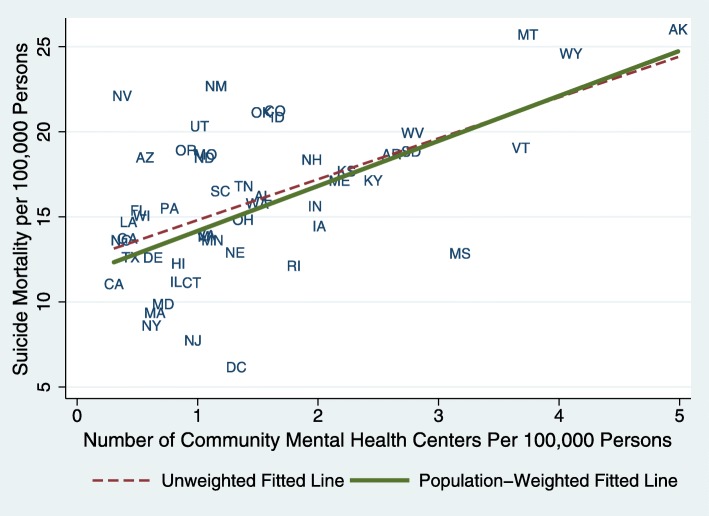
Between-State Associations between Community Mental Health Center Availability and Suicide Deaths per 100,000 Persons. NOTES Data were from 2014 to 2017 National Mental Health Services Survey (N-MHSS) to identify state-level community mental health center status during 2014–2017 and from Centers for Disease Control and Prevention (CDC) Wide-Ranging Online Data for Epidemiologic Research (WONDER) data for suicide rates, identified as intentional self-harm by ICD-10-CM diagnosis codes U03, X60-X71, X72-X74, X75-X84, Y87.0. Each state’s coordinate represents its number of suicide deaths and community mental health centers per 100,000 persons in 2017. The unweighted fitted line was from unadjusted linear regression, giving each state equal weight, while population-weighted fitted line was from population-weighted linear regression, accounting for state population in 2017. A Community Mental Health Center included self-identified community mental health centers and partial hospitalization/day treatment facilities; a community mental health center was defined as a facility that provided any of the following services: 1) outpatient services, 2) 24-h emergency care services, 3) day treatment or other partial hospitalization services, or psychosocial rehabilitation services, and 4) screening for inpatient services to state mental health facilities, and that met applicable licensing or certification requirements for community mental health centers in a state where it is located [7]

Figure 2 graphically depicts the between-state associations between the 2014–2017 state average CMHC availability per 100,000 and state average suicide rate per 100,000. These data indicate that states with more CMHC availability have higher suicide rates.

### Multivariate analysis of community mental health centers and suicide

The results of multivariate time series models are shown as average marginal effects, indicating the estimated annual change in suicide deaths per 100,000 persons associated with a one-unit increase for continuous variables (Table 2). The first model shows a significant positive between-state association between the availability of CMHCs and suicide rates, as one would expect based on Fig. 2 (Table 2; Model 1). In contrast, states with higher supply of hospital-based psychiatric units or with more mental health professionals per capita had lower suicide rates. However, after controlling for time-invariant differences between states (e.g., mental health needs) and underlying time trends (Table 2; Model 2), we estimated that one additional CMHC per 100,000 persons was associated with a decrease in number of suicides (Average Marginal Effects: -0.52, 95% CI − 1.03 to − 0.02; *p* = 0.043). After adding controls for age, race, percent low-income, one CMHC increase per 100,000 persons was associated with a *decrease* of 0.52 suicide deaths per 100,000 persons (− 0.52, 95% CI − 1.08 to 0.03; *p* = 0.066). Additionally, increases in number of hospitals with psychiatric services and mental health professionals were associated with *increases in* state suicide mortality.

**Table 2:** Associations between State-level Mental Health Services Capacity and Suicide Mortality Rates 2014–2017

	Coefficients [95% Confidence Interval]			
	Model 1	Model 2	Model 3	Model 4
	Between-state Model	Add State & Time	Add Age and Race	Add Low-income
State Indicators Included	No	Yes	Yes	Yes
**Number of Community Mental Health Centers Per 100,000 Persons**	2.78 (2.36, 3.20) ***	−0.52 (−1.03, −0.02)*	−0.60 (− 1.09, −0.11)*	**−0.52 (− 1.08, 0.03)§**
**Number of Hospital Psychiatric Care Settings per 100,000 Persons**	−1.70 (−2.54, − 0.85) ***	0.75 (− 0.72, 2.21)	**1.32 (− 0.03, 2.67)§**	1.39 (0.02, 2.75)*
**Number of 100 Mental Health Professionals per 100,000 Persons†**	−0.71 (− 1.26, − 0.16)*	0.10 (− 0.21, 0.41)	−0.10 (− 0.51, 0.30)	−0.10 (− 0.46, 0.26)
**Year (Reference: 2014)**				
2015		0.27 (0.20, 0.34)***	0.52 (0.32, 0.73)***	0.45 (0.27, 0.63)***
2016		0.39 (0.31, 0.48)***	0.91 (0.50, 1.31)***	0.52 (0.17, 0.87)**
2017		0.80 (0.67, 0.93)***	1.67 (1.14, 2.20)***	1.17 (0.69, 1.65)***
**% Persons Below 200% Federal Poverty Level**				−0.09 (−0.14, −0.04)**
**% Population by Age Group**				
Less than 15 Years Old			Ref	Ref
15–24 Years Old			1.02 (0.63, 1.42)***	0.63 (0.21, 1.06)**
25–44 Years Old			−0.15 (− 0.55, 0.25)	−0.94 (−1.34, − 0.53)***
45–64 Years Old			−1.27 (− 1.57, − 0.97)***	−1.82 (−2.41, − 1.23)***
65–74 Years Old			−0.14, − 0.92, 0.64)	−0.41 (− 1.15, 0.32)
75 Years Old or More			−1.50 (− 2.56, − 0.45)**	−1.81 (− 2.53, − 1.10)***
**% Population by Race/Ethnicity**				
Non-Hispanic White			Ref	Ref
Non-Hispanic Black			0.84 (0.73, 0.94)***	0.93 (0.67, 1.18)***
American Indian and Alaska Native			1.45 (−0.74, 10.33)	−0.98 (−10.24, 8.28)
Asian			−1.70 (− 2.28, − 1.12)***	−1.67 (− 2.15, − 1.18)***
Native Hawaiian and Pacific Islander			21.47 (16.58, 26.37)***	22.49 (18.01, 26.96)***
Hispanic			−1.43 (− 1.61, − 1.25)***	−1.41 (− 1.58, − 1.24)***
Two or More Races			− 1.48 (− 1.86, − 1.10)***	−1.42 (− 1.79, − 1.05)***

NOTES Data were from 2014 to 2017 National Mental Health Services Survey (N-MHSS) to identify state-level mental health facility availabilities during 2014–2017 and from Centers for Disease Control and Prevention (CDC) Wide-Ranging Online Data for Epidemiologic Research (WONDER) data for suicide rates, identified as intentional self-harm by ICD-10-CM diagnosis codes U03, X60-X71, X72-X74, X75-X84, Y87.0. Significance at *p*-values of § *p* < .1, * *p* < .05, **p < .01, ***p < .001 were noted for the average marginal effects of suicide mortality rates per a unit increase for continuous variables, switching from the reference group, accounting for intra-state correlation across years. Estimates were derived from multivariate linear regression models, weighted by state population size. †Data on mental health professionals, including psychiatrists, psychiatric technicians, psychiatric aides, clinical, counseling, and school psychologists, all other psychologists, mental health counselors, mental health and substance abuse social workers, were derived from U.S. Bureau of Labor Statistics [22]

To better understand the magnitude of the associated suicide deaths with CMHC changes per capita, we consider the number of suicides that may have been prevented had the number of CMHCs not been reduced over this time period. Considering the population-weighted number of CMHCs decreased from 1.07 per 100,000 in 2014 to 0.90 per 100,000 in 2017 (Appendix Table 3), indicating 0.17 per 100,000 fewer CMHCs nationally. The estimated effect in Model 4 (− 0.52) suggests the change in CMHC would lead to 0.0884 additional suicides per 100,000 (− 0.17× − 0.52 = 0.0884. Given 1.48 per 100,000 more suicides between 2014 and 2017 (15.37 and 16.85 per 100,000, respectively), this accounts for 6.0% of the increase in suicides over the four-year period (0.0884/1.48). With the national increase in 4400 suicide deaths from 2014 (42,773) to 2017 (47,173) [1], this represents 263 additional suicide deaths following the loss of CMHCs (6.0% × 4400 = 262.8).

State-level age, race, and low-income distribution were expected not to change significantly over the four study years and therefore would not be significantly associated with the changes in suicide rates during 2014–2017. Yet, we observed a positive association between increased suicide rates and the higher proportion of individuals in a given state who were Black (0.93, 95% CI 0.67 to 1.18]; *p* < .001) or Native Hawaiian and Pacific islanders (22.49, 95% CI 18.01 to 26.96]; *p* < .001), and a negative association between decreased suicide rates and the higher proportion of the individuals who were Asian (− 1.67, 95% CI -2.15, − 1.18; *p* < .001*),* Hispanic (− 1.41, 95% CI -1.58, − 1.24; *p* < .001), or two or more races (− 1.42, 95% CI -1.79, − 1.05; *p* < .001).

**Fig. 3:**
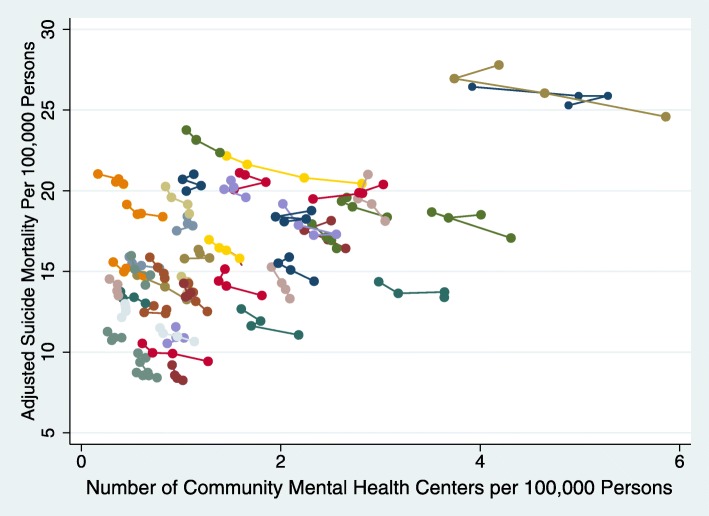
Within-State Associations between Number of Community Mental Health Centers and Suicide Mortality Per 100,000 Persons. NOTE There are 51 states with 3 years of data. Data from 2014 to 2017 National Mental Health Services Survey (N-MHSS) to identify state-level community mental health center status during 2014–2017 and from Centers for Disease Control and Prevention (CDC) Wide-Ranging Online Data for Epidemiologic Research (WONDER) data for suicide rates, identified as intentional self-harm by ICD-10-CM diagnosis codes U03, X60-X71, X72-X74, X75-X84, Y87.0. Predicted suicide mortality rates were estimated from the population-weighted multivariate linear regression model, adjusting for state-level number of mental health facility supply and mental health professionals per 100,000, state-level populations by age, state-level populations by race, state-level populations below 200% federal poverty level, as well as state and year. Connected points signify the same states

To further illustrate the within-state associations between the number of CMHCs per 100,000 persons and suicide rates we find in the final model (i.e., Model 4), we calculated adjusted suicide rates, controlling for hospital-based psychiatric supply, mental health professional supply, age, race, and % low-income in a state, state fixed effects, and year. These adjusted rates have already controlled for underlying time trends in suicide rates, and the average suicide rate in each state over the time period studied. We then plotted the four predicted suicide rates for each state, with a line indicating results for the same state (Fig. 3). This figure demonstrates that, controlling for the underlying increase in suicide trends nationwide, within states, positive (negative) changes in CMHCs are associated with smaller (greater) changes in suicide.

## Discussion

This study documents the changing landscape of CMHCs in the US. During 2014–2017, the number of specialty mental health centers that self-identified as CMHCs decreased by 14% from 3406 to 2920. Despite a national increasing trend in suicide mortality rates for decades, 6 states experienced decreasing suicide rates during this study period, allowing us to examine how changes in state-level CMHCs per capita were associated with such variations. We found the decrease in the availability of CMHCs was associated with an increase in suicide mortality rates. Of the 1.48 per 100,000 increase in the suicide rate from 2014 to 2017, 6% was due to declines in the number of CMHCs, representing 263 suicide deaths. This is just one very limited outcome related to mental health status. The decreasing availability of CMHCs may also be associated with significant increases in other mental health symptoms, constituting significant cumulative effects over time.

Suicide is a multifaceted issue, involving individual, family, community, and social risk factors [23]. Ongoing discussions about mental health care accessibility factors related to suicide have been focused on hospital-based psychiatric supply [3, 4, 6]. Our findings add information regarding the potentially important role of community mental health services on the increasing trend of suicide rates. It is not surprising that CMHCs may have a greater relationship with suicide mortality than psychiatric hospital capacity. A nationwide study in Finland also found a promising association between the presence of CMHCs and lower suicide mortality rates [24]. In the U.S., substantially more patients receive treatment in CMHCs compared to inpatient facilities [7]. In particular, while approximately 370 thousand patients were being treated at psychiatric hospitals or general hospitals with dedicated psychiatric beds (some on an outpatient basis) at one point in time in 2017, more than three times as many individuals (1.3 million) were in treatment at CMHCs [7]. As patients often receive mental health treatment at general hospitals or outpatient facilities with a primary focus on general medical conditions, these facilities may have less access to supportive mental health services for patients with serious mental illness or requiring medications accompanied with greater risk of suicide [25, 26].

One essential supportive mental health component for suicide is a dedicated suicide prevention program. This study found that within-state increases in suicide prevention services in outpatient care settings are critical in reducing suicide mortality rates. Yet, suicide prevention is more complicated than an outpatient mental health facility’s simply offering suicide prevention services. Holistic specialty services such as hotline services, case management, suicide prevention programs, peer support groups, among others, are often beneficial to suicide [27]. CMHCs typically provide a range of specialized services for individuals with serious mental illness, including those who have been discharged from an inpatient mental health facility [7, 28]. In addition to suicide prevention services, other types of supportive services offered at CMHCs – such as psychiatric emergency walk-in services, case management, and crisis intervention treatments – may complement the existing suicide prevention services and facilitate suicide prevention. In 2017, nearly half of CMHCs provide psychiatric emergency walk-in services and over 85% provide case management which helps practitioners meet the needs of clients and their families [7]. More importantly, one half of CMHCs provide suicide prevention services [7]. Other psychosocial services such as 24-h intensive community services, multidisciplinary clinical team approach, supported housing, supported employment and vocational rehabilitation services are more likely to be offered at CMHCs compared to other outpatient mental health facilities, and have been shown to affect patient outcomes [7, 29, 30]. Even minimal connections with a mental health professional post-suicide attempt has been shown to have a protective effect on suicide [31].

It is encouraging to uncover that states with higher, on average, suicide mortality rates had higher CMHCs supply. It may be that states with particularly high mental health needs or suicide rates have historically devoted more resources to mental health treatment, or a greater share of their mental health dollars to CMHCs, relative to other types of prevention or treatment.

For years, CMHCs have faced financial distress due to patients’ payer mix and a lack of funding [7, 9, 10]. Medicaid, state general funds, and federal block grants are the funding sources used by CMHCs [32]. Although patients with serious mental illness have been increasingly covered by Medicaid, about one-third of those served by state mental health authorities did not have Medicaid [33]. Also, the improved access to insurance coverage does not guarantee access to services, as inadequate state funding could leave CMHCs vulnerable to closure, yielding insufficient mental health care infrastructure in a community. For example, from 2010 to 2014, state mental health agency controlled mental health spending in real dollars was generally flat or declining [33]. During this period, the U.S. state mental health agency’s mental health services expenditures (in 2001 dollars) slightly decreased from $26.4 to $26.2 billion [33]. State reductions mental health agency budgets might have exacerbated the financial challenges facing CMHCs, subsequently undermining their abilities to provide specialized care [10, 34].

Among adults with serious mental illness, about one-third received no formal treatment within the past year, and 40% reported unmet need for mental health services [14]. Lack of service use is a substantial problem for even those with the most serious conditions [14]. Among individuals hospitalized with a mental health disorder only 35–53% receive any follow-up within 7 days post discharge [35]. Given that in the 3-month period following discharge from psychiatric hospitalization, patients are at exceptionally high risk for suicide [5], availability of specialized community services is particularly important in ensuring that patients receive follow-up support or referral to appropriate placements.

The shortage of mental health providers, particularly psychiatrists, makes it difficult to ensure adequate community-based services are available to treat mental illness [36, 37]. Although the emphasis on integrating mental health services into primary care, in some cases in conjunction with specialty consultants has increased [38], specialty suicide prevention services in the community mental health care settings still play an important role for individuals with serious mental illness. Prior studies have documented the success of telepsychiatry in response to mental health provider shortages in underserved communities [27, 39–41]. However, the adoption of these systems has not been widespread [42]. Approaches to establishing telemedicine systems for serving serious mental illness should target areas facing the loss of community-based mental health services, as well as those at high risk of losing those services.

This study is not without limitations. While the eligibility criteria for inclusion in the N-MHSS did not change during the study period, these data resulted from self-reported categories of facility types and of care settings provided. Our study is limited in that we focused on the availability of licensed CMHCs and hospital psychiatric services; we excluded from our analysis other potential mental health treatment settings–such as FQHCs, emergency departments, general community health centers, other specialty outpatient settings, residential treatment programs, and home health delivery. Our focus exclusively on CMHCs means that some of our results may be due to changes in classification of facilities. Most notably, partial hospitalization was given a category distinct from other outpatient mental health facilities. In addition, in October 2014, Medicare provided certification requirements for the Medicare-certified CMHC-partial hospitalization program. We mitigated this potential bias by defining a facility that reported as CMHCs or partial hospitalization/day treatment programs as a CMHC. Our findings proved that changes in the supply of suicide prevention services at the CMHC settings play an important role in state suicide mortality; CMHCs had the highest rates of offering suicide prevention services across all outpatient mental health facility types.

Because CMHCs were not separately identified in the N-MHSS until 2014, this study used a short time period. Several limitations preclude us from establishing a causal link between change in CMHC availability and suicide mortality. The data on CMHCs was only available at the state level – more granular data would have increased our confidence, the effects found in this study were causal, and such analyses should be pursued in future work. The short study duration does not allow us to include state-specific time trends in our models. Reductions in the number of CMHCs may be associated with other unmeasured factors that are causing changes in the suicide rate. For example, changes in state mental health needs, public mental health funding and its implicated mental health services consolidation are likely to be associated with both CMHC availability and the availability of other mental health prevention and treatment programs. Likewise, increases in state suicide rates may be due to factors that are also rendering reductions in CMHC supply, such as state economic conditions.

## Conclusions

This study provides important information about the changing landscape of CMHCs in the U.S. and of its association with suicide deaths. The decrease in the number of CMHCs per capita in a state was associated with a within-state increase in suicide mortality. In the era of hospital psychiatric supply shortages, the decreasing trend of CMHCs highlights an urgent need to develop practical and accessible treatment models for seriously mentally ill patients who are at greatest risk of suicide, especially those in areas with decreasing CMHC supply.

## Supplementary information

**Additional file 1 Appendix Table 1**. Characteristics of Outpatient Mental Health Care Settings by Facility Type in 2014–2017. **Appendix Table 2** Change in Mental Health Care Settings and Mental Health Professionals per 100,000 Persons by State, 2014–2017. **Appendix Table 3** State Characteristics by Data Year

## Data Availability

The datasets used and/or analyzed during the current study are publicly available. National Mental Health Services Survey (N-MHSS) https://www.samhsa.gov/data/data-we-collect/n-mhss-national-mental-health-services-surve y[43]. The Centers for Disease Control and Prevention (CDC) Wide-Ranging Online Data for Epidemiologic Research (WONDER) https://wonder.cdc.gov /[44].

## Abbreviations

CMHC: Community mental health center
OECD: Organization for Economic Co-operation and Development
CMHA: Community Mental Health Act
N-MHSS: National Mental Health Services Survey
CDC: Centers for Disease Control and Prevention
WONDER: Wide-Ranging Online Data for Epidemiologic Research
ICD: International Classification of Diseases
CM: Clinical Modification;
CI: Confidence intervals
