# Supplementary material for: Changes in community mental health services availability and suicide mortality in the US: a retrospective study
Source: BMC Psychiatry. 2020 Apr 25;20:188. doi: 10.1186/s12888-020-02607-y (PMC7183673; doi:10.1186/s12888-020-02607-y)
Supplement: Supplementary file 1 — Additional file 1 Appendix Table 1. Characteristics of Outpatient Mental Health Care Settings by Facility Type in 2014–2017. Appendix Table 2 Change in Mental Health Care Settings and Mental Health Professionals per 100,000 Persons by State, 2014–2017. Appendix Table 3 State Characteristics by Data Year [file 12888_2020_2607_MOESM1_ESM.docx]

**Appendix Table 1. Characteristics of Outpatient Mental Health Care Settings by Facility Type in 2014-2017**

|  | **Community Mental Health Centers/Partial Hospitalization/Day Treatment Settings** | | | | **Outpatient Mental Health Centers** | | | | **Multiple Care Settings** | | | |
| --- | --- | --- | --- | --- | --- | --- | --- | --- | --- | --- | --- | --- |
|  | **2014** | **2015** | **2016** | **2017** | **2014** | **2015** | **2016** | **2017** | **2014** | **2015** | **2016** | **2017** |
| **Number of Facilities** | 3406 | 3218 | 3024 | 2920 | 4493 | 5037 | 4771 | 4587 | 552 | 497 | 397 | 387 |
| **Care Settings** |  |  |  |  |  |  |  |  |  |  |  |  |
| 24-hour hospital inpatient services | 1.3% | 1.9% | 1.1% | 1.2% | 0.5% | 0.5% | 0.3% | 0.0% | 6.2% | 14.1% | 6.8% | 4.1% |
| 24-hour residential | 1.3% | 1.3% | 1.0% | 1.2% | 0.6% | 0.5% | 0.4% | 0.3% | 44.4% | 44.5% | 42.8% | 48.6% |
| Partial hospitalization/day treatment | 17.5% | 21.0% | 17.5% | 23.0% | 9.9% | 7.7% | 5.7% | 9.5% | 58.3% | 51.9% | 49.9% | 53.2% |
| Outpatient | 96.3% | 91.6% | 92.4% | 92.7% | 98.0% | 99.1% | 99.7% | 99.8% | 80.8% | 90.9% | 92.9% | 92.3% |
| **Ownership** |  |  |  |  |  |  |  |  |  |  |  |  |
| Private for-profit | 7.3% | 8.6% | 8.4% | 8.4% | 21.4% | 19.4% | 19.0% | 19.2% | 18.8% | 21.9% | 20.2% | 20.9% |
| Private non-profit | 72.8% | 70.5% | 71.6% | 72.0% | 61.8% | 63.1% | 64.0% | 64.3% | 69.7% | 68.6% | 72.5% | 71.6% |
| Public agency or department | 19.9% | 20.9% | 20.0% | 19.6% | 16.8% | 17.6% | 17.0% | 16.6% | 11.4% | 9.5% | 7.3% | 7.5% |
| **Accept for Treatment by Age** |  |  |  |  |  |  |  |  |  |  |  |  |
| All Age | 62.0% | 60.7% | 61.4% | 62.4% | 55.9% | 54.7% | 56.4% | 57.2% | 34.8% | 43.3% | 47.1% | 45.2% |
| Children (12 or younger) | 74.8% | 73.5% | 73.6% | 74.7% | 72.5% | 72.5% | 74.7% | 74.7% | 61.6% | 62.2% | 66.2% | 65.1% |
| Adolescents (13-17) | 77.4% | 76.1% | 76.0% | 76.9% | 76.1% | 76.3% | 77.7% | 77.7% | 68.7% | 70.6% | 74.1% | 72.4% |
| Young adults (18-25) | 93.9% | 91.8% | 92.7% | 92.7% | 91.7% | 91.6% | 92.4% | 93.0% | 88.2% | 91.5% | 96.0% | 94.8% |
| Adults (26-64) | 88.2% | 87.8% | 88.3% | 87.6% | 87.0% | 85.1% | 84.8% | 87.6% | 73.4% | 83.3% | 83.6% | 83.2% |
| Seniors (65 or older) | 85.2% | 84.8% | 85.7% | 85.5% | 81.4% | 80.3% | 80.0% | 85.5% | 65.9% | 75.3% | 76.3% | 75.5% |
| **Payment Acceptance** |  |  |  |  |  |  |  |  |  |  |  |  |
| Medicare | 76.6% | 78.6% | 79.8% | 80.0% | 67.8% | 67.2% | 67.4% | 67.7% | 58.3% | 63.6% | 62.2% | 58.9% |
| Medicaid | 95.2% | 96.0% | 96.2% | 96.6% | 89.2% | 89.1% | 90.9% | 91.4% | 87.1% | 90.9% | 90.7% | 88.6% |
| Cash or self-payment | 86.5% | 89.3% | 90.4% | 90.5% | 85.8% | 84.1% | 83.8% | 84.2% | 81.5% | 84.7% | 88.4% | 90.7% |
| Private health insurance | 81.0% | 83.3% | 85.0% | 86.0% | 78.0% | 78.1% | 78.8% | 79.0% | 78.8% | 80.3% | 83.4% | 81.7% |
| Other payments (e.g., no charge,  other public payment) | 64.0% | 60.7% | 63.0% | 64.6% | 50.3% | 50.9% | 51.8% | 50.0% | 50.5% | 51.5% | 52.9% | 47.3% |
| **Service Provision** |  |  |  |  |  |  |  |  |  |  |  |  |
| Suicide prevention services | 72.7% | 55.6% | 62.0% | 66.5% | 61.5% | 42.0% | 47.8% | 50.3% | 66.1% | 46.3% | 48.9% | 52.5% |
| Crisis intervention treatment Team | 60.7% | 62.7% | 64.3% | 66.7% | 37.9% | 36.7% | 36.7% | 37.1% | 52.4% | 50.5% | 50.4% | 47.6% |
| Case management | 79.1% | 75.6% | 78.9% | 84.1% | 57.8% | 55.2% | 57.9% | 59.3% | 73.7% | 68.6% | 72.0% | 72.9% |
| Chronic disease/illness management | 18.1% | 16.3% | 17.7% | 19.2% | 14.2% | 13.3% | 14.7% | 14.5% | 19.0% | 20.5% | 18.6% | 18.6% |
| Integrated primary care services | 19.2% | 17.8% | 20.9% | 23.5% | 16.7% | 16.5% | 18.0% | 18.2% | 24.5% | 24.3% | 22.9% | 25.6% |
| Psychosocial rehab services | 51.6% | 44.1% | 49.2% | 51.3% | 36.3% | 30.6% | 34.4% | 35.0% | 52.2% | 41.4% | 46.6% | 50.9% |

NOTE Data on facility characteristics were derived from 2014-2017 National Mental Health Services Survey (N-MHSS).

**Appendix Table 2. Change in Mental Health Care Settings and Mental Health Professionals per 100,000 Persons by State, 2014-2017**

|  | Number of Community Mental Health Centers/Partial Hospitalization/Day Treatment Settings Per 100,000 Persons | | | Number of Mental Health Professionals Per 100,000 Persons | | |
| --- | --- | --- | --- | --- | --- | --- |
|  | 2014 | 2017 | % Change 2014-2017 | 2014 | 2017 | % Change 2014-2017 |
| Nationwide | 1.67 | 1.45 | -13.17% | 173.16 | 222.29 | 28.37% |
| AK | 4.89 | 3.92 | -19.84% | 244.32 | 297.38 | 21.72% |
| AL | 1.61 | 1.44 | -10.81% | 136.31 | 136.42 | 0.08% |
| AR | 3.07 | 2.66 | -13.26% | 117.65 | 161.10 | 36.93% |
| AZ | 0.82 | 0.46 | -44.38% | 116.17 | 195.40 | 68.20% |
| CA | 0.4 | 0.26 | -34.24% | 147.77 | 194.15 | 31.39% |
| CO | 1.53 | 1.59 | 3.74% | 180.18 | 263.77 | 46.39% |
| CT | 0.86 | 0.95 | 10.18% | 249.12 | 324.68 | 30.33% |
| DC | 1.06 | 1.01 | -4.84% | 437.1 | 383.30 | -12.31% |
| DE | 0.86 | 0.73 | -15.38% | 152.84 | 178.81 | 16.99% |
| FL | 0.78 | 0.48 | -38.29% | 116.42 | 147.35 | 26.57% |
| GA | 0.64 | 0.39 | -38.57% | 70.61 | 104.61 | 48.15% |
| HI | 1.06 | 0.56 | -47.13% | 148.64 | 166.72 | 12.16% |
| IA | 2.09 | 1.91 | -8.74% | 140.32 | 151.64 | 8.06% |
| ID | 2.81 | 1.46 | -48.18% | 144.39 | 177.64 | 23.03% |
| IL | 1.13 | 0.79 | -30.18% | 140.83 | 186.45 | 32.40% |
| IN | 2.33 | 2.08 | -10.52% | 136.43 | 157.65 | 15.55% |
| KS | 2.65 | 2.51 | -5.44% | 164.6 | 205.62 | 24.92% |
| KY | 2.56 | 2.31 | -9.67% | 123.03 | 181.63 | 47.63% |
| LA | 0.62 | 0.32 | -48.35% | 121.73 | 163.52 | 34.33% |
| MA | 0.67 | 0.57 | -15.15% | 436.45 | 423.48 | -2.97% |
| MD | 1.27 | 0.61 | -51.86% | 128 | 193.15 | 50.90% |
| ME | 2.33 | 2.02 | -13.26% | 254.87 | 208.85 | -18.06% |
| MI | 1.06 | 1.00 | -5.30% | 132.09 | 165.12 | 25.01% |
| MN | 1.26 | 1.08 | -14.61% | 161.44 | 275.97 | 70.95% |
| MO | 0.96 | 1.06 | 10.75% | 169.21 | 224.42 | 32.63% |
| MS | 3.64 | 2.98 | -18.06% | 222.44 | 238.26 | 7.11% |
| MT | 5.86 | 4.19 | -28.52% | 378.09 | 402.67 | 6.50% |
| NC | 0.37 | 0.28 | -23.71% | 139.88 | 164.11 | 17.32% |
| ND | 0.54 | 0.93 | 71.61% | 116.3 | 153.56 | 32.04% |
| NE | 0.74 | 1.41 | 90.03% | 152.01 | 179.68 | 18.20% |
| NH | 2.03 | 2.31 | 13.73% | 101.75 | 232.35 | 128.35% |
| NJ | 1.02 | 0.91 | -10.73% | 183.82 | 204.54 | 11.27% |
| NM | 1.39 | 1.05 | -24.20% | 186.04 | 240.41 | 29.23% |
| NV | 0.42 | 0.17 | -60.29% | 78.19 | 118.08 | 51.01% |
| NY | 0.76 | 0.55 | -27.08% | 196.59 | 258.09 | 31.29% |
| OH | 1.81 | 1.44 | -20.39% | 137.4 | 182.78 | 33.03% |
| OK | 1.65 | 1.50 | -9.03% | 135.12 | 214.71 | 58.90% |
| OR | 1.08 | 0.84 | -21.77% | 209.56 | 279.76 | 33.50% |
| PA | 0.84 | 0.69 | -18.19% | 246.34 | 337.82 | 37.14% |
| RI | 2.18 | 1.60 | -26.41% | 234.08 | 284.06 | 21.35% |
| SC | 1.03 | 1.17 | 14.01% | 64.77 | 116.63 | 80.07% |
| SD | 3.05 | 2.87 | -5.75% | 94.94 | 188.58 | 98.63% |
| TN | 1.59 | 1.28 | -19.46% | 129.94 | 170.04 | 30.86% |
| TX | 0.4 | 0.44 | 9.52% | 116.15 | 153.76 | 32.38% |
| UT | 1.05 | 1.13 | 7.46% | 159.71 | 200.20 | 25.35% |
| VA | 1.04 | 1.03 | -1.24% | 244.41 | 289.85 | 18.59% |
| VT | 4.31 | 4.01 | -6.99% | 288.88 | 585.26 | 102.60% |
| WA | 1.26 | 1.53 | 21.10% | 116.55 | 198.09 | 69.96% |
| WI | 0.64 | 0.50 | -21.81% | 132.7 | 230.87 | 73.98% |
| WV | 2.32 | 3.03 | 30.55% | 242.66 | 286.92 | 18.24% |
| WY | 4.79 | 3.97 | -17.11% | 152.36 | 157.08 | 3.10% |

NOTES Number of community mental health centers per 100,000 in each state year were calculated using the 2014-2017 National Mental Health Services Survey (N-MHSS). Data on mental health professionals, including psychiatrists, psychiatric technicians, psychiatric aides, clinical, counseling, and school psychologists, all other psychologists, mental health counselors, mental health and substance abuse social workers, were derived from U.S. Bureau of Labor Statistics [[42](#_ENREF_42)].

**Appendix Table 3. State Characteristics by Data Year**

|  | **Population-Weighted Mean (Standard Deviation)** | | | |
| --- | --- | --- | --- | --- |
|  | 2014 | 2015 | 2016 | 2017 |
| **Number of Community Mental Health Centers/Partial Hospitalization/Day Treatment Settings Per 100,000 Persons** | 1.07 (0.77) | 1.00 (0.73) | 0.94 (0.68) | 0.90 (0.70) |
| **Number of Hospital Psychiatric Care Settings per 100,000 Persons** | 0.56 (0.23) | 0.57 (0.23) | 0.57 (0.23) | 0.53 (0.22) |
| **Number of Mental Health Professionals per 100,000 Persons**§ | 1.57 (0.63) | 1.57 (0.53) | 1.62 (0.55) | 2.04 (0.67) |
| **Number of Outpatient Settings Providing Suicide Prevention Services per 100,000 Persons** | 3.19 (1.71) | 3.16 (1.65) | 2.99 (1.55) | 2.86 (1.44) |
| **Number of Inpatient Settings Providing Suicide Prevention Services per 100,000 Persons** | 1.42 (0.70) | 1.37 (0.64) | 1.26 (0.60) | 1.19 (0.54) |
| **% Population Below 200% Federal Poverty Level** † | 34.96 (5.22) | 33.73 (5.10) | 29.86 (4.33) | 28.06 (4.67) |
| **% Population by Age Group** ‡ |  |  |  |  |
| Less than 15 Years Old | 19.17 (1.59) | 19.01 (1.58) | 18.87 (1.56) | 18.73 (1.54) |
| 15-24 Years Old | 13.80 (0.63) | 13.64 (0.65) | 13.47 (0.67) | 13.28 (0.68) |
| 25-44 Years Old | 26.34 (1.43) | 26.33 (1.44) | 26.35 (1.47) | 26.47 (1.54) |
| 45-64 Years Old | 26.18 (1.44) | 26.14 (1.42) | 26.08 (1.39) | 25.91 (1.35) |
| 65-74 Years Old | 8.28 (0.98) | 8.58 (1.00) | 8.86 (1.03) | 9.11 (1.03) |
| 75 Years Old or More | 6.22 (1.01) | 6.30 (1.00) | 6.38 (1.00) | 6.50 (1.01) |
| **Population Distribution by Race and Ethnicity** ‡ |  |  |  |  |
| Non-Hispanic White | 62.06 (15.35) | 61.69 (15.39) | 61.28 (15.44) | 60.77 (15.51) |
| Non-Hispanic Black | 12.40 (8.11) | 12.42 (8.11) | 12.45 (8.11) | 12.48 (8.11) |
| American Indian and Alaska Native | 0.74 (1.51) | 0.74 (1.51) | 0.74 (1.51) | 0.74 (1.52) |
| Asian | 5.24 (4.43) | 5.37 (4.47) | 5.49 (4.52) | 5.65 (4.59) |
| Native Hawaiian and Pacific Islander | 0.17 (0.65) | 0.17 (0.65) | 0.18 (0.65) | 0.18 (0.65) |
| Hispanic | 16.88 (12.91) | 17.04 (12.94) | 17.24 (12.98) | 18.05 (13.26) |
| Two or More Races | 2.51 (1.65) | 2.57 (1.65) | 2.62 (1.67) | 2.13 (1.36) |

NOTES Data on state-level hospital-based psychiatric services, community mental health center status, and facility suicide prevention services status during 2014-2017 were from 2014-2017 National Mental Health Services Survey (N-MHSS) and from Centers for Disease Control and Prevention (CDC) Wide-Ranging Online Data for Epidemiologic Research (WONDER) data for suicide rates, identified as intentional self-harm by ICD-10-CM diagnosis codes U03, X60-X71, X72-X74, X75-X84, Y87.0. † Data on population below 200% federal poverty level were from American Community Survey Poverty Levels, 2014-2017. ‡Data on population distribution of age and race/ethnicity are from U.S. Census Projected Population by Age, Sex, Race, and Hispanic Origin, 2014-2017. § Data on mental health professionals, including psychiatrists, psychiatric technicians, psychiatric aides, clinical, counseling, and school psychologists, all other psychologists, mental health counselors, mental health and substance abuse social workers, were derived from U.S. Bureau of Labor Statistics [[42](#_ENREF_42)].
